# IL-6 as a Mediator of Platelet Hyper-Responsiveness

**DOI:** 10.3390/cells14110766

**Published:** 2025-05-22

**Authors:** Connor Elliot Webb, Jordan Vautrinot, Ingeborg Hers

**Affiliations:** School of Physiology, Pharmacology and Neuroscience, Biomedical Sciences Building, University Walk, Bristol BS8 1TD, UK; connor.webb@bris.ac.uk (C.E.W.); jordan.vautrinot@bris.ac.uk (J.V.)

**Keywords:** interleukin-6, platelets, hyper-responsiveness, thrombo-inflammation, thrombosis

## Abstract

Interleukin-6 (IL-6) is a pleiotropic cytokine with critical roles in immune regulation, inflammation, and haematopoiesis. While its functions in host defence and tissue repair are well established, accumulating evidence suggests that IL-6 also can directly and indirectly modulate megakaryocyte and platelet biology. This review examines the mechanistic basis supporting IL-6-mediated platelet hyper-responsiveness, in addition to its effect on megakaryopoiesis and thrombopoiesis in thromboinflammatory disease states. We discuss how IL-6-mediated trans-signalling may sensitizes platelets to activation, and that this may be exclusive to glycoprotein VI (GPVI) stimulation due to Janus kinase (JAK)–signal transducer 2 crosstalk, in addition to other mechanisms that may contribute to priming platelets. We further highlight clinical evidence linking IL-6 to thrombotic complications in cardiovascular disease and infection (e.g., COVID-19 and sepsis). Given the emerging interest in IL-6-targeting therapies as anti-inflammatory and anti-thrombotic agents, a thorough understanding of how IL-6 can drive platelet responsiveness is crucial.

## 1. Introduction

In 1931, MacKay [1] described platelets—small, discoid-shaped, anuclear blood cells—as being of minor importance’ to clotting, as well as a mere building block to be integrated into a thrombus, all of which was orchestrated solely by the *intrinsic* clotting cascade. This doctrine was thoroughly challenged during the 1960s. Gaarder, et al. [2] demonstrated that red-blood-cell-derived adenosine-5’-phosphate (ADP) could induce platelet aggregation. Haslam [3] later determined that the addition of a potent agonist, such as thrombin, to platelets would result in sufficient ADP release to seemingly self-perpetuate platelet aggregation [4]. Most notably, in the past two decades, platelets have exhibited a wide range of non-canonical roles beyond thrombosis and haemostasis. Through the synthesis, release and capture of inflammatory mediators, in addition to cell-to-cell interactions, platelets are key contributors to inflammation, and thereby potential insidious participants, inadvertently promoting inflammation in various pathologies [5,6,7].

Indeed, platelets are part of the ‘central hub’ of inflammation, with their behaviour being modulated by circulating compounds, as well as frequent endothelial and immune cell interactions [7]. Emerging research has demonstrated that the release of cytokines attributed to inflammatory pathologies can also perturb normal platelet behaviour, resulting in aberrant, hyperresponsive phenotypes that can, in turn, intensify systemic inflammation and elevate the risk of thrombotic events. This emerging concept is referred to as ‘thrombo-inflammation’, the nexus in which thrombosis and inflammation meet, and has been associated with multiple pathologies, including cardiovascular diseases (CVD), as well as infectious diseases (including sepsis and COVID-19), and autoimmune conditions [8].

The pleiotropic cytokine interleukin (IL)-6 has recently been implicated as an immunothrombotic agent and is often associated with an increased risk of life-threatening complications [9,10,11,12]. IL-6 interacts with platelets in multiple dimensions, increasing thrombopoiesis and accelerating thrombus development [13,14,15,16]. IL-6 can also ‘prime’ platelets by lowering the threshold of platelet activation, resulting in platelet hyper-responsiveness [17,18]. Furthermore, impeding IL-6 signalling through pharmacological blockade, such as the use of tocilizumab (TCZ), is being investigated for its potential to reduce thrombotic complications [19,20]. Despite this, the relationship between IL-6 and platelets, including how IL-6 drives platelet hyper-responsiveness, is poorly defined, with contradicting findings in vivo and in vitro. This review will speculate on multiple potential mechanisms by which IL-6 can drive platelet hyper-responsiveness and evaluate the existing experimental evidence to support this phenomenon. It will also examine the relationship between the dysregulation of the IL-6 axis and perturbed platelet behaviour in multiple diseases.

## 2. Primary Mechanisms of Platelet Activation

To appreciate the underlying mechanisms of platelet priming and the potential mechanisms of IL-6 priming, it is essential to delineate the difference between priming and activation. Platelet activation is generally initiated by a soluble or bound agonist activating its cognate receptor [21]. This can be due to a single agonist, such as thrombin or collagen, or summative, due to the multitude of transmembrane receptors expressed on the platelet membrane. The resulting intracellular cascades converge intracellularly, triggering the activation of α_Iib_β_3_ integrins (‘inside-out’ signalling), allowing for fibrinogen binding to the newly active conformation, resulting in platelet aggregation and thrombus formation [22]. If the concentration and potency of the respective agonist are sufficient, the contents of α- and dense-granules are also secreted. This results in the expression of granule-bound membrane proteins (i.e., PECAM, CD9 and GLUT-3), some of which are exclusively expressed upon granule secretion (most notably, P-selectin), as well as secondary mediators to promote paracrine activation and aggregation (i.e., fibrinogen and ADP) [23,24,25]. Over 300 soluble factors are released following granule secretion, and many of these promote auto- and paracrine potentiation by binding constitutively expressed transmembrane receptors [26]. This highly orchestrated response, fine-tuned by the synthesis and release of thromboxane A_2_ (TxA_2_), allows for an ‘all-or-nothing’ response when challenged by a ‘strong’ agonist, or the synergism of weak agonists [27]. The most frequently described ‘strong’ agonists are thrombin and collagen.

### 2.1. PAR-Mediated Signalling and Activation

**Figure 1:**
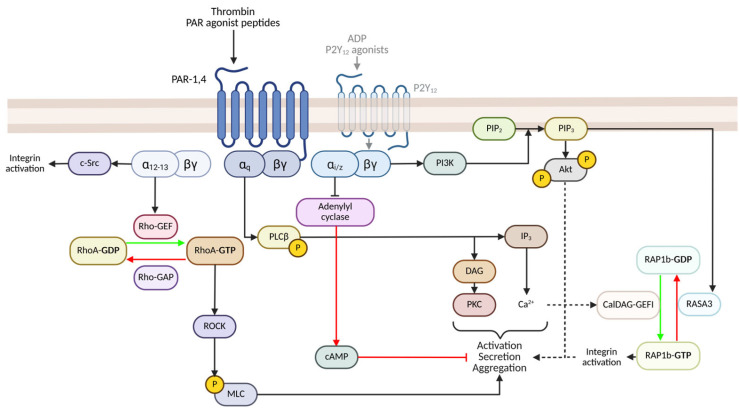
Simplified PAR-mediated signalling cascade in platelets. Thrombin or PAR agonist peptides cleave the extracellular N-terminus to trigger GPCR signalling. Gα_q_ promotes PLCβ phosphorylation and downstream PKC and Ca^2+^ mobilisation; Gα_12/13_ promotes phosphorylation of MLC and supports integrin activation; Gα_i_ impairs cAMP-mediated inhibition, and the release of the βɣ subunit promotes PI3K/Akt activation, as well as RAP1b-mediated integrin activation. Not all pathways (i.e. MAPK) have been illustrated for brevity; refer to the main text for acronyms and further details. Red and green arrows indicate inhibitory and activatory processes, respectively. Created in BioRender, agreement number: US289BKVN2. Webb, C. (2025) https://BioRender.com/yiqn1gw (accessed on 12 May 2025).

Thrombin is generated through the sequential activation of the coagulation cascade, serving as a critical serine protease that catalyses the conversion of fibrinogen to fibrin. In addition to its role in fibrin formation, it is a potent platelet agonist, leading to platelet activation. Human platelets exhibit at least three thrombin receptors: two G-protein coupled protease-activated receptors (PAR), PAR-1 and -4, and the abundantly expressed glycoprotein Ib-IX (GPIb-IX). The simplified schema of PAR-mediated signalling is illustrated in detail in Figure 1. Thrombin irreversibly cleaves the N-terminus of the PAR receptor, resulting in a tethered peptide ligand that binds to and activates the PAR receptor. PAR-4 requires a higher thrombin concentration and is considered significantly slower in initiating platelet activation than PAR-1. Therefore, PAR-1 is regarded as the ‘primary’ thrombin receptor [28]. The receptor confirmation changes activate associated intracellular G-proteins such as Gα_q_ and Gα_12,13_.

Gα_q_ is critical for platelet activation and signals through phospholipase Cβ (PLCβ), which catalyses the hydrolysis of phosphatidylinositol biphosphate (PI4,5P_2_) to inositol 1,4,5-trisphosphate (IP_3_) and diacylglycerol (DAG). The former, IP_3_, contributes to the mobilization of Ca^2+^, while DAG activates PKC [29,30]. The Ca^2+^ flux induces granule secretion and, through CalDAG-GEFI, stimulates RAP1b-GDP conversion to RAP1b-GTP, thereby promoting Talin-mediated integrin α_Iib_β_3_ activation [23,24]. Gα_12/13_, potentially synergising with Gα_q_, stimulates Rho-guanine nucleotide exchange factor (Rho-GEF), promoting the conversion of RhoA-GDP to RhoA-GTP [31,32,33]. RhoA-GTP activates Rho-associated protein kinase (ROCK), inactivating MLC phosphatase, as well as phosphorylation of myosin light-chain (MLC), resulting in a shape change [34]. Gα_12/13_ also triggers the binding of c-Src with the integrin β_3_ tail, promoting early integrin α_Iib_β_3_ outside-in signalling and cell spreading [35,36].

Thrombin can also indirectly activate the G_i_ signalling pathway by stimulating dense granule secretion, leading to ADP release, and subsequent activation of the G_i_-coupled P2Y_12_ receptor (Figure 1). Gα_i_ coupling inhibits adenylyl cyclase, impairing cAMP synthesis and preventing cAMP-mediated inhibition. The relinquishing of G_βɣ_ subunit promotes the activation of PI3K, catalysing the conversion of PI(4,5)P_2_ to PI(3,4,5)P_3_ [37]. The effector Akt is activated downstream of PI(3,4,5)P_3_, but it additionally recruits the Rap and Ras GTPase-activating protein (GAP) RASA3 to the membrane surface [38]. PI(3,4,5)P_3_-mediated recruitment of RASA3 to the plasma membrane inhibits it GAP activity towards RAP1b, facilitating sustained RAP1b activation and integrin α_Iib_β_3_ activation [39,40]. The combined activation of Gα_q_, Gα_12/13_ and Gα_i_ signalling pathways ultimately results in platelet activation, granule secretion and aggregation [37].

### 2.2. GPVI-Mediated Signalling and Activation

**Figure 2:**
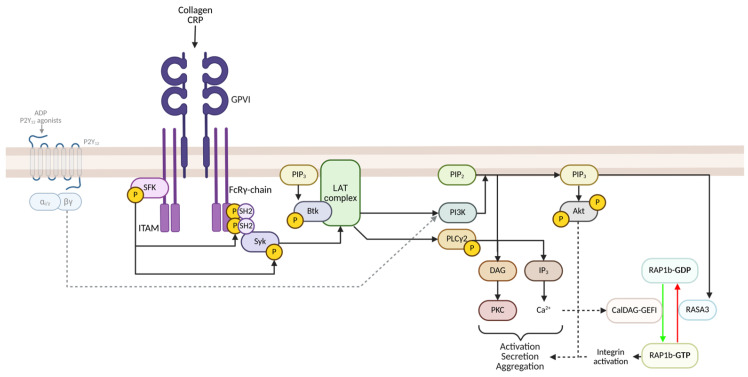
Simplified GPVI-mediated signalling cascade in platelets. Activation of the GPVI receptor by collagen or collagen-related peptide (CRP) triggers Src family kinases (SFK), which phosphorylate the ITAM region on the associated FcRɣ chain. This promotes the recruitment and activation of Syk, initiating the assembly of the LAT signalling complex. This complex facilitates the recruitment of PI3K and BTK, ultimately resulting in the activation of PLCɣ2. Subsequent activation of PKC and Ca2+ mobilization lead to platelet activation, granule secretion and platelet aggregation. Not all pathways (i.e. MAPK) have been illustrated for brevity; refer to the main text for acronyms and further details. Red and green arrows indicate inhibitory and activatory processes, respectively. Created in BioRender, agreement number: VC289BL06P. Webb, C. (2025) https://biorender.com/wj3a8et (accessed on 12 May 2025).

The stimulation of the GPVI receptor initiates activation of Src Family Kinases (SFKs), leading to tyrosine phosphorylation of the immunoreceptor tyrosine-based activation motif (ITAM) region of the FcRɣ chain. This results in the recruitment of Spleen tyrosine kinase (Syk) via its Src Homology 2 (SH2) domain to the ITAM region, leading to its phosphorylation and activation [41,42]. Syk subsequently phosphorylates the adaptor protein Linker for the activation of T cells (LAT). This leads to the assembly of a signalling complex on the adaptor protein LAT, which forms the LAT signalosome and brings together multiple downstream effectors. These include phosphoinositide 3-kinase (PI3K), which generates the lipid second messenger PI(3,4,5)P₃ at the membrane. PIP₃ is essential for recruiting Bruton’s tyrosine kinase (BTK), leading to its phosphorylation and activation (Figure 2). Activated BTK acts as a keystone modulator of PLCɣ2 activation and is essential in GPVI-mediated activation [43,44]. 

PLCɣ2 mediates the activation of PKC through DAG, and IP_3_-mediated increases in intracellular Ca^2+^ in a similar manner to PLCβ in GPCR-mediated activation. PLCɣ2 is associated with prolonged and sustained signalling and is required for GPIb- and platelet ITAM receptor activation and, therefore, platelet functioning [45]. Furthermore, ADP, released from the dense granules, binds to P2Y_12_ receptors, and through autocrine Gα_i_ signalling, further promotes platelet activation by inhibiting adenylyl cyclase and activating PI3K [46,47].

## 3. Mechanisms of Platelet Priming

Emerging evidence suggests that circulating ‘non-canonical’ agonists, called ‘primers’, sensitise platelets to become more responsive to traditional agonists but do not induce activation independently. Platelet priming reduces the threshold for platelet activation, ultimately promoting a hyper-responsive phenotype. These primers include a wide spectrum of hormones, cytokines, and chemokines, among other compounds (see Table 1). Priming can also be included in physical means, such as mechanical changes (i.e., increased shear stress). Platelets exhibit a multitude of cognate receptors, most of which are exclusive to a specific primer; however, recently, some receptors have emerged as promiscuous (i.e., the binding of PF4 to the TPO receptor, c-Mpl) or can be co-opted (i.e., the cleavage of PAR-1 by MMP-2 utilising integrin α_Iib_β_3_ as a cofactor). The initial cascade can vary substantially primer-to-primer; however, most typically induce PI3K (i.e., Gas6, IGF-1 and TPO) and/or MAPK (i.e., TPO) downstream. Primers utilising PI3K can help potentiate secretion, promote integrin α_Iib_β_3_ activation, as well as increase TxA_2_ synthesis following activation by an agonist [48]. Primers that utilise GPCRs, such as epinephrine and the corresponding α_2A_ receptor, can additionally utilise Gα_i/z_ signalling to impair adenylyl cyclase, decreasing cAMP levels and lowering the threshold to activation [49]. Note, however, that this is not sufficient to fully initiate platelet activation, due to the multitude of pathways that need to be activated simultaneously, in addition to the inhibition of cAMP synthesis, to allow for full activation. There is notable redundancy between the effector cascades, with some primers (i.e., PF4 and TPO) utilising several pathways; however, this dichotomy is likely due to limited reporting, rather than exclusivity.

### 3.1. Synergism Between Circulating Primers

In vivo, platelets are exposed to multiple primers, which can act in tandem to potentiate specific agonist-induced activation. This can be due to synergism between primer–receptor interactions converging intracellularly, utilization of a promiscuous receptor, or a non-interactive, summative effect [80]. For example, thrombopoietin (TPO) and insulin-like growth factor-1 (IGF-1) both utilise the downstream phosphoinositide 3-kinase (PI3K) pathway [50,78,81,82]. Agonism of the TPO receptor by c-MPL (c-myeloproliferative leukaemia protein) activates Janus kinase 2 (JAK2), resulting in tyrosine phosphorylation of signal transducer and activator of transcription (STAT) proteins and the regulatory p85 subunit of PI3K. The tyrosine phosphorylation of p85 liberates multiple catalytic p110 subunits, with two of these isoforms, p110β and p110γ, coordinating multiple synergistic actions, including α-granule secretion, RAP1b/integrin α_Iib_β_3_ activation, MAPK activation, and thromboxane A2 (TxA2) formation and release [79]. IGF-1-mediated priming is similarly coordinated. The binding of IGF-1 to insulin-like growth factor-1 receptor (IGF-1R, or CD221), or IGF-1R/insulin receptor hybrids, results in phosphorylation of insulin substrate-1 and -2 (IRS-1,-2), which subsequently recruit and dock the p85 subunit, freeing the p110α and p110β PI3K isoforms to trigger downstream Akt phosphorylation and activation [82]. Exposing washed platelets to TPO and IGF-1 simultaneously can reverse the inhibition induced by ASA and AR-C66096 in vitro [83].

Platelet factor 4 (PF4) is a highly cationic chemokine, stored in platelet α-granules, and is released upon platelet activation [84]. Anti-PF4 antibodies have been heavily attributed as the mechanistic agent in vaccine-induced immune thrombotic thrombocytopenia (VITT) and heparin-induced (immune) thrombotic thrombocytopenia (HITT); however, the direct interaction is poorly defined [85]. PF4 has been known as a platelet primer, potentiating thrombin-induced activation, and an agonist (at sufficient concentrations), but the mechanism has only recently been elucidated [64,66]. PF4 primes platelets through the TPO receptor, c-Mpl, utilising multiple downstream pathways including JAK2/STAT3 and PI3K, similar to TPO and unique pathways such as STAT5a/b and p38 MAPK [66]. PF4 binds to a site adjacent to TPO on c-Mpl, in part disrupting the binding of TPO to c-Mpl; therefore, synergism between PF4 and TPO, especially at lower, physiological concentrations, while not demonstrated, is a possibility.

### 3.2. Primers and ‘Anti-Platelet Resistance’

Multiple studies have demonstrated that individually or in combination, primers can partially reverse or completely rescue platelet function following incubation with antiplatelet agents, such as aspirin and/or P2Y12 inhibitors. For example, succinate, a Krebs cycle intermediate induced by metabolic stress, can act as an agonist at high concentrations and enhance adenosine diphosphate (ADP)-induced aggregation at lower concentrations [86]. ADP responsiveness of platelets following incubation with P_2_Y_1_ or P_2_Y_12_ inhibitors was restored following co-stimulation with ADP and succinate. Dose-dependent administration of succinate can also partially or fully reverse the effect of ASA administration on arachidonic acid (AA)-induced platelet aggregation [87]. Veninga, et al. [78], using flow cytometry, noted that succinate also primed platelets to thrombin-related activator peptide-6 (TRAP-6, PAR-1 agonist) and collagen-related peptide (CRP, GPVI agonist). Blair, et al. [83] previously demonstrated a similar phenomenon when using TPO, IGF-1, and epinephrine. The authors noted that adding IGF-1, TPO, or epinephrine to washed platelets treated with ASA and/or AR-C66096 (ARC) rescued PAR-1 and GPVI-mediated aggregation. However, although physiological concentrations of these individual primers did not affect ASA/ARC-mediated inhibition of in vitro thrombus formation, when combined, the capacity to form a thrombus was fully restored. These findings support the notion that primers may depend on synergism to counteract anti-platelet therapy [21].

This has resulted in primers emerging as potential novel targets to mediate platelet hyper-responsiveness and/or augment traditional antiplatelet agents, especially in cardiovascular diseases. For example, Gas6, the product of the growth arrest-specific 6 gene, is a vitamin K-dependent plasma protein and has been implicated as a potential target since its elucidation as a primer in 2001 [53,88,89]. Gresele, et al. [90] outlined the potential of inhibiting Gas6 and other primers using neutralising antibodies or proteases. The group also developed MMP-2 nanobodies to investigate the possibility of anti-MMP-2 as a therapeutic agent that could reduce platelet hyper-responsiveness [91,92]. JAK inhibitors have also been suggested as possible mediators. Ruxolitinib successfully blocks TPO and PF4-driven hyper-responsiveness, and other agents have been evaluated in system models to potentially impair platelet homeostasis in vivo [66,93]. However, few therapeutics explicitly targeting primers to mediate platelet responsiveness have reached the pharmaceutical market; however, therapies targeting IL-6, a proposed primer, have recently emerged as a potential strategy to target thrombo-inflammation [94].

## 4. Interleukin-6 Structure and Signalling

IL-6 has been recognised as a highly pleiotropic cytokine that influences a wide range of biological processes, including innate and adaptive immunity, haematopoiesis, metabolism, tissue remodelling and most importantly, thrombosis and coagulation [9,95]. Under homeostatic conditions, circulating IL-6 levels remain low; however, infections, tissue damage, or autoimmune exacerbations can trigger marked increases in IL-6 concentrations, stimulating hepatocytes to produce acute-phase proteins such as C-reactive protein (C-RP), fibrinogen, and serum amyloid A [96]. This acute-phase reaction facilitates pathogen isolation, stabilises injured tissues, and enhances immune defences, reflecting IL-6’s indispensable role in host protection. Persistent elevation of IL-6 can promote chronic inflammation and accelerate disease-associated pathophysiology in conditions such as atherosclerosis, rheumatoid arthritis, and infection-related complications [97,98].

### 4.1. IL-6 and IL-6-Associated Receptors Structure and Expression

IL-6 has long been of interest in haemostasis, ranging from its role in megakaryopoiesis to suggestions of it as a direct activator of platelets themselves. IL-6 was initially identified as B-cell stimulatory factor 2 (BSF-2) in the mid-1980s and is known for its capacity to induce terminal differentiation of B cells into immunoglobulin-secreting plasma cells [99]. Other proteins, such as interferon-β2 (IFN-β2), hepatocyte-stimulating factor, and plasmacytoma growth factor, were also noted in short succession as homologues of BSF-2 [99,100]. Ultimately, BSF-2 and its homologues were designated as ‘interleukin (IL)-6’ by Sehgal, et al. [101]. Yamasaki, et al. [102] successfully isolated and cloned human IL-6. The associated receptors, interleukin(IL)-6 receptor (IL-6R) and glycoprotein 130 (gp130), were also isolated and cloned [103].

IL-6 and other IL-6 family cytokines are distinguished by four α-helical bundles, with several Ig-like and fibrinogen type III domains. IL-6R is described as a two-component class I cytokine receptor, with a 90-kDa IL-6 binding protein (the IL-Rα-chain), with corresponding Ig-like and fibrinogen type III domains, which co-opts gp130 for signal transduction [104,105,106]. This association is essential due to the absence of a tyrosine kinase domain on the α-domain [107]. The majority of cord-blood-derived CD34^+ve^ cells express gp130, but IL-6Rα is only expressed in 30–50% of these cells, with a notable absence in megakaryocyte progenitors [108]. IL-6Rα expression and the stage of megakaryopoiesis are described as negatively correlated; following commitment to the megakaryocyte lineage, the α-chain expression peaks, then progressively diminishes until it is undetectable on the platelet membrane [109]. This is described in a similar manner for another IL-6 family cytokine, IL-11R [109]. It is unknown whether newly synthesised ‘young’ platelets still exhibit some level of IL-6R, which is then lost shortly after.

**Figure 3:**
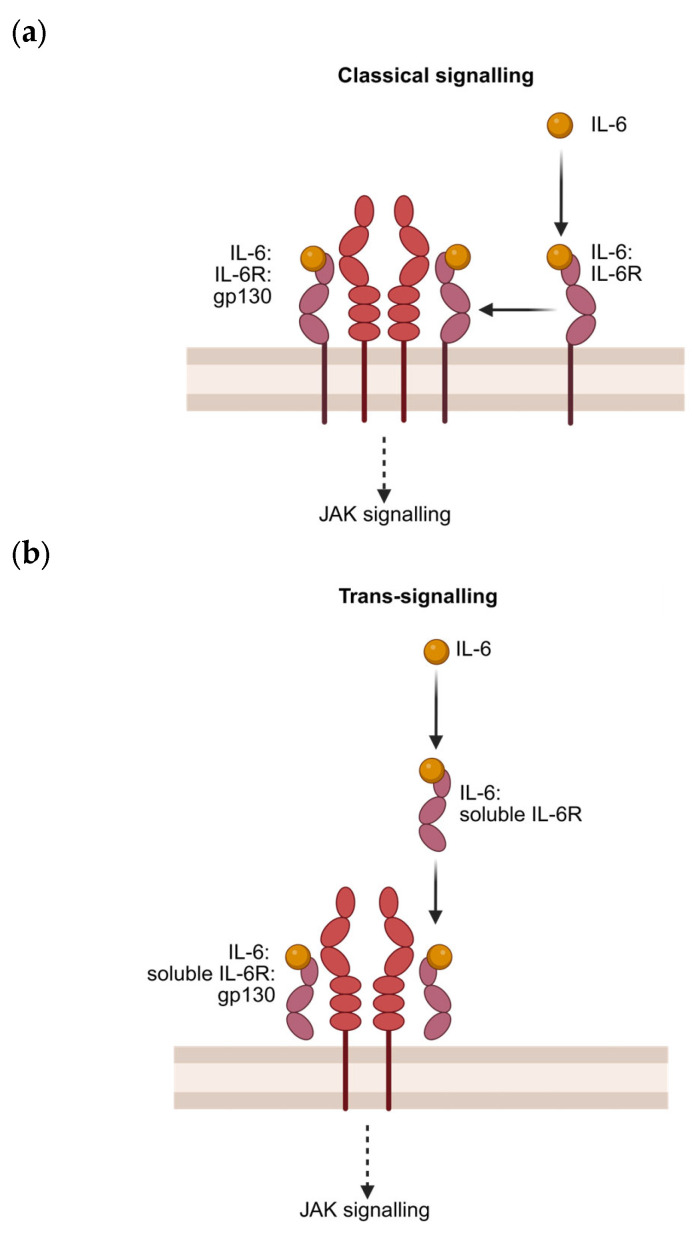

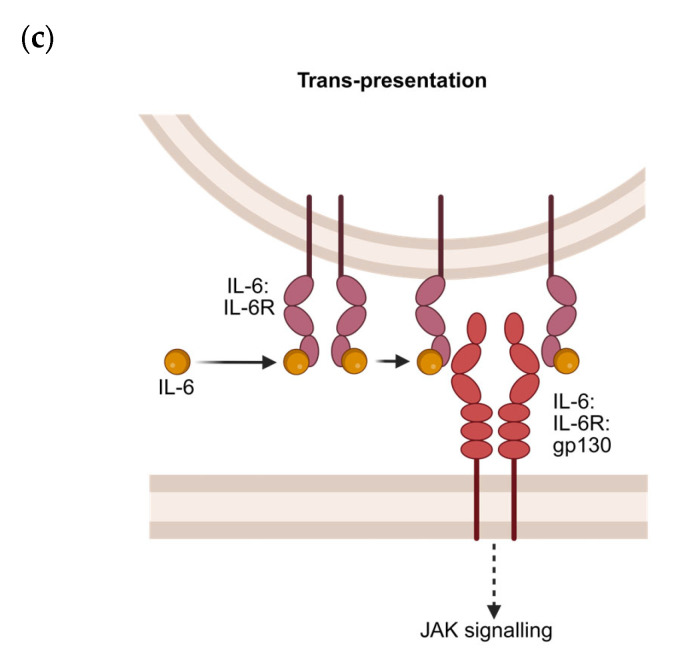
The three mechanisms of IL-6-mediated signalling, classical, trans-signalling and trans-presentation, are simplified. (**a**) In classical signalling, IL-6 binds to the IL-6R, homodimerises, and then binds to gp130 to create a competent, hexameric signalling complex. (**b**) In trans-signalling, IL-6 binds to soluble IL-6R before forming a similar signalling complex with gp130 on the membrane of IL-6R-naïve cells. (**c**) In trans-presentation, IL-6 binds to IL-6R on a separate cell, then binds parallel to a gp130 complex on an IL-6R-naïve cell to trigger signalling. Gp130, glycoprotein 130; IL-6, interleukin-6; IL-6R, interleukin-6 receptor. Created in BioRender, agreement number: SL289BLJNP. Webb, C. (2025) https://BioRender.com/ioe0ndo (accessed on 12 May 2025).

A Disintegrin and Metalloproteinase (ADAM)-17-mediated shedding of IL-6Rα has been speculated; however, Marta, et al. [107] found minimal changes in soluble IL-6R concentration when an ADAM-17 inhibitor (TAPI, TNF-α protease inhibitor) was incubated with a platelet suspension. Further studies have shown that circulating soluble IL-6R is from both ADAM-10 and ADAM-17 acting as sheddases, the former being constitutive and the latter being inducible. Soluble IL-6R concentrations are also in part bolstered by alternative mRNA splicing [110,111]. It is likely that the lack of IL-6R expression on platelets is the combined product of reduced expression in mature megakaryocytes and continual ADAM-mediated sheddase activity, supported by the lack of transcriptional activity in platelets. Regardless of this, IL-6 can utilise three distinct mechanisms of IL-6 signalling, one of which is independent of IL-6R expression, all with differential outcomes. They are referred to as (a) classical or canonical, (b) trans-signalling, and (c) trans-presentation (Figure 3).

### 4.2. Canonical Signalling

Canonical IL-6 signalling is initiated when soluble IL-6 binds to the membrane-bound IL-6 receptor (also known as CD126), forming a binary complex (IL-6R: IL-6R, ‘site I’) (Figure 3a). This binary complex then interacts with the cytokine-binding region (CBR) of membrane-bound glycoprotein 130 (also known as CD130), creating an intermediate composite epitope (‘site II’). This tertiary structure dimerises, utilising the outermost extracellular Ig-like domain (IgD) on gp130 (‘site III’) to create the final hexameric signalling complex (IL-6: IL-6R: gp130) [112]. This assembly initiates downstream phosphorylation of Janus kinases (JAK1, JAK2, and TYK2) via the intracellular domain of gp130 [99]. These phosphorylated kinases subsequently phosphorylate multiple tyrosine sites on gp130, promoting the activation of STAT proteins, most notably STAT3, which can then induce the transcription of genes associated with acute-phase protein production, immune modulation, and cell survival [100].

### 4.3. Trans-Signalling and Presentation

In 1989, the Kashimoto group demonstrated a novel mechanism by which IL-6 binds to soluble IL-6R (sIL-6R), which subsequently binds to gp130 (Figure 3b) [113]. Mackiewicz, et al. [114] successfully demonstrated that this complex could induce acute-phase protein production from human hepatocytes, similarly to canonical IL-6 signalling. Rose-John and Heinrich [115] coined the novel mechanism ‘trans-signalling’, and Stoyan, et al. [116] demonstrated that trans-signalling allows for IL-6-mediated signalling in cells without IL-6R expression. The ubiquitous expression of gp130 and the existence of IL-6 trans-signalling explain the pleiotropic properties exhibited [117]. Importantly, trans-signalling allows IL-6 to signal in platelets lacking the IL-6R, as discussed later in this review, highlighting the essential role of soluble IL-6R in its impact on platelet function. Recently, a third signalling mechanism, trans-presentation, was discovered, in which IL-6 bound to one cell’s IL-6R can bind to a receiver cell’s gp130 to facilitate cell–cell signalling (Figure 3c) [118].

### 4.4. Comparing the Inflammatory Effects Based on Signalling Mechanism

IL-6 is a pivotal cytokine in inflammation, operating primarily through two distinct signalling pathways: classical (canonical) and trans-signalling. Classical signalling occurs when IL-6 binds to the membrane-bound IL-6 receptor, which is restricted to immune cells such as macrophages, T cells, and hepatocytes, leading to anti-inflammatory and homeostatic effects via the JAK-STAT3 pathway [119]. Conversely, trans-signalling occurs when IL-6 interacts with the soluble IL-6 receptor, enabling the cytokine to activate mgp130-expressing cells that lack IL-6R, such as endothelial cells and fibroblasts, thereby driving chronic inflammation [120]. This distinction is crucial in autoimmune and inflammatory diseases, as trans-signalling promotes Th17 differentiation while suppressing regulatory T cells, exacerbating conditions such as rheumatoid arthritis and inflammatory bowel disease [99]. The therapeutic implications are significant, as global IL-6 blockade (e.g., tocilizumab) may suppress both beneficial and deleterious effects of IL-6. In contrast, selective inhibition of trans-signalling via soluble gp130 Fc (gp130Fc, olamkicept) preserves classical IL-6 functions necessary for tissue repair [121]. Understanding the mechanistic divergence between classical and trans-signalling is essential for developing targeted therapies that mitigate pathological inflammation without compromising essential immune functions.

### 4.5. Modulation of IL-6 and IL-6 Signalling

The IL-6 axis is regulated through two primary mechanisms: regulation of the synthesis and release of IL-6, and modulation of circulating IL-6 signalling. The acute-phase response (APR) is an example of this mechanism. APR is a systemic phenomenon that accompanies inflammation, in which IL-6, interleukin(IL)-1β (IL-1β), and tumour necrosis factor-α (TNF-α) synergistically cooperate to mitigate potential infection and further tissue injury [122,123,124]. IL-6 is responsible for producing a vast spectrum of acute-phase reaction proteins (APPs), including serum C-RP and amyloid A, from hepatocytes, including the upregulation of IL-6 [125]. This feed-forward loop also occurs in other cells, such as CD4+ T cells, which systematically contributes to the elevation of circulating IL-6 [95,126]. Following the restoration of tissue homeostasis, these cells subsequently produce cytokine signalling 3 (SOCS3) protein, which inhibits multiple JAK proteins as well as ADAM-mediated IL-6R shedding, disrupting the feed-forward loop and promoting the sequestering of serum sIL-6R [110]. Unsurprisingly, dysregulation of this system, which allows for sustained IL-6 synthesis and release, has been implicated in multiple inflammatory and autoimmune conditions [127,128].

The mechanism of IL-6 signalling is also heavily modulated. Müller-Newen, et al. [129] postulated that the naturally occurring formation of soluble gp130 (gp130) and soluble IL-6R (gp130: sIL-6R) complexes is the biological mechanism to deliberately ‘trap’ IL-6 in a ternary complex, effectively buffering all forms of IL-6 signalling. Conversely, Jostock, et al. [130] demonstrated that this tertiary complex (induced by the addition of recombinant gp130) inhibits gp130-mediated signalling in BAF/3 cells. However, when the experiment was performed using BAF/3 cells expressing both gp130 and IL-6R, the addition of recombinant soluble gp130 did not impede IL-6 signalling. Therefore, this demonstrates that soluble gp130 inhibits IL-6 signalling that requires IL-6R, or gp130 solely inhibits IL-6 trans-signalling. Ultimately, the balance between classical and trans-signalling is highly dependent on the ratio of IL-6R to gp130, with higher molar ratios promoting trans-signalling, and vice versa [119,131].

## 5. Effects of IL-6 on Megakaryocytes and Platelets

IL-6 has been proposed as a platelet primer. There is indeed convincing evidence to indicate that IL-6 can increase platelet function in vivo, but in vitro findings have been more variable. To fully appreciate how IL-6 can independently prime platelet function, it is essential to delineate the direct and indirect effects of IL-6 on haemopoietic stem cells (HSCs), megakaryocytes and platelets.

### 5.1. Megakaryopoesis and Thrombopoesis

**Figure 4:**
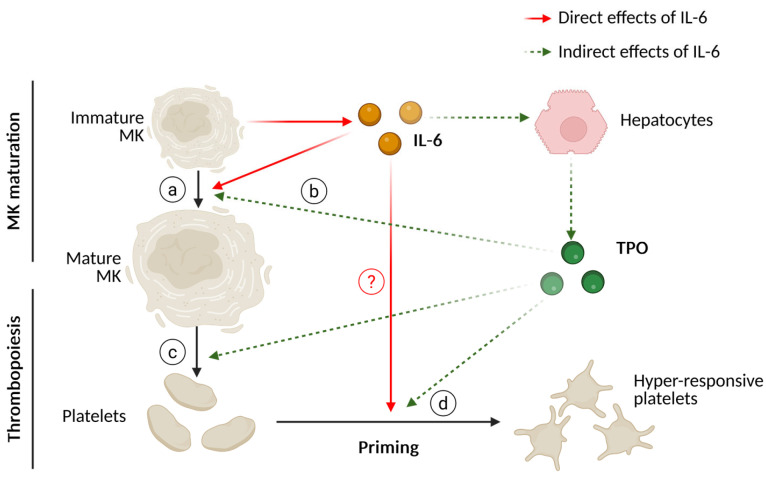
The direct and indirect effects of IL-6 on megakaryocyte maturation, thrombopoiesis, and platelet priming. (a) IL-6 contributes to and sensitises megakaryocytes in an autocrine loop, (b) IL-6 stimulates TPO release from hepatocytes, which in conjunction with other cytokines, drive megakaryocyte maturation, (c) the increase in circulating TPO drives thrombopoiesis and the release of newly synthesised, hyper-responsive platelets, (d) circulating TPO also primes circulating platelet populations. Due to the complexity, the potential effect of IL-6 as a platelet primer is obscured. MK, megakaryocyte; IL-6, interleukin-6; TPO, thrombopoietin. Created in BioRender, agreement number: BD289BLDMQ. Webb, C. (2025) https://BioRender.com/25hodoi (accessed on 12 May 2025).

The predominant mechanism of IL-6 signalling is highly dependent on the expression, or absence, of IL-6Rα. IL-6Rα is not significantly expressed in megakaryocyte-dedicated HSCs, with only 30–50% of cord-blood-derived CD34^+ve^ cells expressing any level of IL-6Rα, with minimal effects on lineage commitment [108,132,133,134]. However, both megakaryoblast and immature megakaryocyte lines (such as cord-blood-derived megakaryoblasts, or the MEG-01 cells, respectively) comprehensively express IL-6Rα, responding to both classical and trans-IL-6-signalling. This is supported by early experimental studies demonstrating that IL-6 could stimulate colony growth of lineage-restricted megakaryocytic and erythroid precursors [135]. Recombinant IL-6 studies demonstrated that while IL-6 can independently promote differentiation in immature megakaryocytes, interleukin-3 (IL-3) is required to meaningfully increase IL-6-mediated megakaryopoiesis [136]. Therefore, IL-6 likely augments the effect of circulating cytokines, such as IL-3, stem cell factor (SCF) and TPO, to accelerate colony formation, poly-ploidisation and maturation (Figure 4a) [137,138,139,140,141]. IL-6 also increases the rate of thrombopoiesis through two mechanisms: firstly, the increased rate of megakaryopoiesis increases the population of competent, mature megakaryocytes, and secondly, IL-6 triggers the secretion of TPO from hepatocytes, indirectly and directly contributing to thrombopoiesis (Figure 4b,c) [142,143].

IL-6 administration in vivo results in an efflux of ‘young’—newly synthesised—platelets. Young platelets are notoriously hyper-responsive, due to increased receptor density and granular content [144]. Furthermore, these platelets are heavily shaped by their circulating environment, which can promote the development of a procoagulant (or ‘COAT’, COllagen And Thrombin) phenotype [145]. Platelets isolated from dogs who underwent subcutaneously administered IL-6 over a ten-day period exhibited a hyper-response thrombin phenotype [146]. A follow-up study demonstrated that only the newly synthesised (thiazole orange-positive, TO^+ve^) platelets exhibited hyper-responsiveness to thrombin [15]. This was supported by Burstein, et al. [147], which documented that the administration of IL-6 increased the sensitivity of dog platelets to thrombin, but additionally augmented plasma von Willebrand Factor (VWF) and plasma fibrinogen. TPO, in and of itself, can act as a primer; however, in washed platelets isolated from cancer patients undergoing recombinant IL-6 therapy, their platelets still demonstrate significantly enhanced ex vivo aggregation (Figure 4d) [83,148,149,150,151]. These findings, taken together, suggest that IL-6 primes platelet function indirectly through enhancing TPO release and the production of young, hyper-responsive platelets.

### 5.2. Soluble IL-6R Is Required for Platelet Priming

Despite early studies noting that the effect of IL-6 on platelet responsiveness is likely to be indirect, more recent studies suggest that IL-6 can also directly affect platelet function, but this requires the presence of sIL-6R. Majka, et al. [152] utilising human gel-filtered platelets, demonstrated that TPO stimulated the phosphorylation of multiple downstream cascades (notably, MAPK p42/44, AKT, STAT-3, and STAT-5), whereas IL-6, as well as other IL-6 family cytokines (IL-11, LIF, or OSM) failed to do so. Isolating and washing platelets may inadvertently have prevented IL-6 trans-signalling through the removal of sIL-6R. Indeed, Senchenkova, et al. [153] found that incubating whole human blood with IL-6, resulted in a significant enhancement in integrin α_IIb_β_3_ activation and P-selectin expression following stimulation with convulxin (a GPVI receptor agonist). Further evidence for the requirement of sIL-6R comes from studies where washed platelets were incubated with IL-6:sIL-6R complexes in a 1:1 molar ratio. Zhou, et al. [18] incubated an excess of IL-6 and sIL-6R in PRP and was able to detect the presence of IL-6 on the surface of platelets within ten minutes at room temperature. Replicating these conditions with washed platelets, the authors observed significant STAT3 phosphorylation, in addition to amplification of GPVI-mediated STAT3 phosphorylation in a concentration-dependent manner [18]. Together, these studies suggest that IL-6-mediated priming of platelet function requires the presence of sIL-6R to allow trans-signalling to occur. Further work is required to confirm that results can be replicated using physiological IL6 concentrations in whole blood and PRP.

### 5.3. IL-6 Priming May Be Limited to GPVI-Mediated Signalling

Despite preserving the integrity of sIL-6R, some studies do not exhibit IL-6-mediated priming. For example, Oda, et al. [16] reported that incubation of platelet-rich plasma (PRP) with IL-6 did not affect epinephrine-induced platelet activation. The choice of an agonist may be of importance, as studies that utilize GPVI agonists, such as collagen or convulxin, demonstrate consistent IL-6 priming, while studies utilising PAR or GPCR agonists, such as TRAP-6 (PAR-1), thrombin (PAR-1 and 4) or epinephrine (α_2A_), fail to do so. Notably, Senchenkova, et al. [153] found that the incubation of human platelets with IL-6 primed GPVI-mediated activation (via convulxin), but not PAR-mediated activation (via thrombin). There is a sole exemption in which IL-6 primed Ca^2+^ ionophore-mediated platelet activation [17,154]. However, these studies also observed direct IL-6-mediated platelet activation, a phenomenon that has not been replicated.

One potential explanation for IL-6 priming to be limited to GPVI-mediated activation is the crosstalk between the gp130-associated JAK2 and the GPVI receptor. JAK2 is involved in GPVI-mediated platelet signalling, with genetic loss-of-function (LOF) or inhibition of JAK2 [and/or STAT3] significantly impairing GPVI, but not thrombin-mediated platelet activation and aggregation [18,155,156,157,158]. Although previous studies have asserted that thrombin stimulation of human platelets also results in JAK2 phosphorylation [159,160]; more recent work by Moore et al. [79] provided evidence to the contrary. A thrombin-stimulated phosphorylated band was indeed present in NP40 JAK2 immunoprecipitants; however, this band disappeared when platelets were extracted in the more stringent RIPA buffer, whilst leaving the TPO-stimulated phosphorylated JAK2 band intact. Furthermore, the thrombin-stimulated phosphorylated band did not align with the subsequent JAK2 re-probe [79]. JAK2 is thus involved in GPVI, but not thrombin-mediated platelet signalling and activation [158].

**Figure 5:**
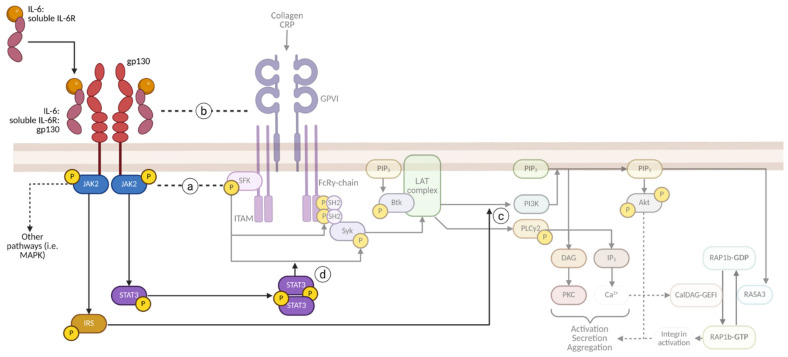
Proposed mechanism of IL-6-mediated priming of GPVI-mediated platelet activation. (a) Crosstalk between gp130 and GPVI via JAK2, (b) close proximity of gp130 and GPVI in lipid rafts (<10 nm), (c) stimulation and amplification of PI3K/Akt, and (d) stimulation and amplification of Syk and PLCɣ2 by STAT3 dimers. Note, P2Y_12_ is not shown for brevity; refer to the main text for acronyms and further details. Created in BioRender, agreement number: HV28AM8B1J. Webb, C. (2025) https://BioRender.com/bynpcyk (accessed on 12 May 2025).

Further evidence for potential cross-talk between IL6 and GPVI comes from proximity studies in human platelets. Utilising fluorescence resonance energy transfer (FRET) and complementary in vitro experiments, Houck, et al. [161] found that gp130 was colocalised and in close proximity (< 10 nm) to GPVI in lipid rafts on human platelets (Figure 5b). Furthermore, the authors noted that downstream gp130 signalling, ergo IL-6-mediated priming, does stimulate JAK2 phosphorylation, which is shared with the GPVI signalling cascade [158]. Utilising a variety of inhibitors and lipid raft-disrupting compounds, the authors determined that the physical proximity of GPVI and gp130 promoted the cross-activation of the collagen-GPVI-Syk and IL-6-JAK2-STAT3 signalling cascades. It is unknown whether PAR, or other pertinent receptors, share this proximity and whether this is why PAR stimulation is unaffected by IL-6 priming. If IL-6-mediated priming is limited to GPVI-mediated activation, this may in part explain the previous findings. Further research is necessary to elucidate whether a discrepancy exists.

### 5.4. IL-6 May Potentiate Multiple Intracellular Cascades Following Platelet Activation

In human vascular cell lines, classical IL-6 signalling does not induce Akt or ERK1/2 phosphorylation; however, trans-signalling results in strong AKT Ser473 and ERK1/2 Thr202/Tyr204 phosphorylation [162]. Some studies have also recorded downstream ERK1/2 amplification and/or activation following IL-6 exposure, such as Bongartz, et al. [163], who demonstrated that IL-6 signalling can amplify late-stage MAPK pathway activation in murine embryonic fibroblasts through Gab1 [164,165]. Although direct evidence in platelets is lacking, the PI3K/Akt and MAPK cassette is highly conserved and tightly controlled; therefore, this may elicit a similar downstream result in platelets (see Figure 5c) [166]. However, it is important to note that neither PI3K nor MAPK is sufficient to independently activate platelets, but their effectors are tied to essential activatory mechanisms [167]. MAPK activation promotes agonist-mediated granule secretion and integrin α_IIb_β_3_-mediated clot retraction [168]. In TPO primed platelet, the phosphorylation and activation of ERK1/2 promotes TxA_2_ synthesis, potentiating priming platelet activation and aggregation [79]. Akt1-3 is involved in granule secretion, integrin α_IIb_β_3_ activation and aggregation in platelets [169]. Furthermore, phosphorylation of Akt also contributes to the regulation of mitochondrial and glycolytic metabolism and the regulation of the multifaceted Thr/Ser kinase, glycogen synthase kinase 3 (GSK-3) [170,171,172]. IL-6 may further promote GPVI-Syk activation through STAT3 scaffolds. STAT3 exhibits non-transcriptional effects in platelets, acting as a scaffold for downstream spleen tyrosine kinase (Syk) and therefore, phosphatidylinositol-specific phospholipase Cγ2 (PLCγ2) activation, which may contribute to potentiating GPVI-mediated signalling following activation (Figure 5d) [18]. STAT3 can promote cross-talk between intracellular cascades as a pro-inflammatory intermediate repository; therefore, other primers associated with inflammation may synergise with IL-6 to summate its effect on GPVI [18].

## 6. Evidence of IL-6-Mediated Platelet Hyper-Responsiveness in Disease

The pleiotropic potential of IL-6 has solidified it as a “keystone cytokine” that mediates the immune system, inflammation, and haemostasis [173]. IL-6 expression and release are primarily mediated by the pro-inflammatory cytokines, tumour necrosis factor-alpha (TNF-α) and interleukin-1-beta (IL-1β), but can be triggered through a myriad of mechanisms, including, but not limited to, the release of prostaglandins and adipokines, as well as damage-associated molecular pattern (DAMP) and Toll-like receptor (TLR) pathway activation [174]. IL-6 is expressed and released from a multitude of cells, including immune-mediated cells, fibroblasts, and endothelial cells, in response to different stimuli [175]. Due to the complexity of IL-6 signalling and the mechanisms by which it exerts its effect on megakaryocytes and platelets, it is hard to ascertain and delineate whether these changes are due to direct platelet priming or indirect mechanisms. Regardless, disruption of the IL-6 axis (including IL-6R and gp130), and in turn, the production of acute phase cytokines, has been implicated in multiple chronic inflammatory conditions, including rheumatoid arthritis (RA), systemic lupus erythematosus (SLE), and psoriasis [176].

### 6.1. Cardiovascular and Thrombotic Diseases

In 1997, Braunwald [177], pulling data from the Framingham Heart Study and Scandinavian Simvastatin Survival Study, noted that despite recent advances, approximately half of all patients with coronary heart disease (CHD) did not exhibit any of the known risk factors at the time of sampling (i.e., hypercholesterolaemia, hypertension, and obesity) [178,179]. In the same year, Ridker, et al. [180] measured the baseline plasma C-RP concentration among men who later experienced ischaemic stroke or MI and demonstrated that C-RP predicted the risk of future thrombotic events. The same group later replicated these findings in a female cohort, demonstrating that high-sensitivity C-RP was the strongest univariate predictor of cardiovascular events, but also noted that elevated IL-6 increased the risk of future cardiovascular events, regardless of lipid levels [181]. Finally, the group successfully demonstrated the link between atherosclerosis and inflammation in the pivotal Canakinumab Anti-Inflammatory Thrombosis Outcomes Study (CANTOS). The inhibition of IL-1β through canakinumab significantly lowered the rates of adverse cardiovascular events without changes in any other known risk factors (such as those outlined by Braunwald [177] above) [182,183]. Inflammation and thrombosis, and by extension, CVD, are intricately linked and partly mediated by platelets, whose behaviour is heavily shaped by their surrounding environment [184,185].

Atherosclerosis is the principal cause of CVD and is initially characterised by the deposition and retention of apolipoprotein (Apo) B-containing lipoproteins in the arteries’ inner layers. These Apo-B lipoprotein plaques accumulate, promoting immune cell recruitment, proliferation of fibrous tissues, and invasion of the surrounding smooth muscle. Calcium deposition and connective tissue formation cause sclerosis, promoting plaque formation and rupture [186]. The role of IL-6 in worsening atherosclerosis has been implicated in numerous experimental murine studies, human cohorts, and genetic studies [183,187]. Recently, the Trial to Evaluate Reduction in Inflammation in Patients with Advanced Chronic Renal Disease Utilising Antibody-Mediated IL-6 Inhibition (RESCUE) trial successfully demonstrated that the inhibition of IL-6 via ziltivekimab substantially reduced atherosclerosis-associated inflammation and thrombosis with minimal effect on platelet counts [187,188].

A recent systematic review conducted by Katkenov, et al. [189] reported that elevated IL-6 levels were significantly associated with myocardial infarction (MI), peripheral arterial disease (PAD) and heart failure (HF). Moreover, a meta-analysis concluded that there was a significant association between IL-6 levels and an increased risk of adverse cardiovascular events. Conversely, Ritschel et al. [190] noted that IL-6 and gp130 levels did not correlate with the future risk of an adverse event in ST-elevation myocardial infarction (STEMI); however, patients with high levels of sIL-6R exhibited an increased risk of both adverse events and mortality. An elevated sIL-6R to gp130 ratio has previously been attributed to an increase in IL-6 trans-signalling, the more pro-inflammatory mechanism of IL-6 signalling, and the only signalling mechanism that can prime platelets [119,131].

Single-nucleotide polymorphisms (SNPs) that perturb the IL-6 signalling axis have been causally associated with CVD. Using Mendelian randomisation principles, in which genetic variants can be assessed for their causal relationship with observed phenotypes, Swerdlow, et al. [191] conducted a meta-analysis of 34 studies and described an IL6R rs7529229 SNP, which resulted in an increase in sIL-6R and a significantly decreased risk of coronary heart disease events [129]. However, this discrepancy has been attributed to the increased buffering of IL-6 signalling (through soluble IL-6R and gp130 binding).

Elevated IL-6 has been implicated in residual platelet hyper-responsiveness and stent thrombosis (ST) after percutaneous coronary intervention (PCI). Müller, et al. [190], utilising a cohort of patients with symptomatic coronary artery disease (CAD) treated with PCI, demonstrated a significant association between baseline IL-6, C-RP and regulated activation, normal T-cell expressed and secreted protein (RANTES) and AA- and ADP-induced multiple electrode aggregometry (in whole blood). Analysing data from the Korea Stent Thrombosis Registry, Hwang, et al. [192] found that the highest IL-6 quartile was associated with a high risk of developing ST. Finally, using multivariate regression analysis, Gremmel, et al. [193] found that IL-6 was independently associated with platelet integrin α_IIb_β_3_ and P-selectin expression, as well as increased concentrations of PLA, following PCI with stent implantation.

Colitis inflammation, in part mediated by IL-6, has been associated with platelet hyper-responsiveness and an increase in circulating platelet–leukocyte aggregates (PLA) and activated platelets, possibly contributing to cardiovascular disease [194]. Senchenkova, et al. [13], utilising an IL-6 double-knockout (DKO) murine model (IL-6^−/−^), explore how IL-6 mediates the relationship between colonic inflammation and platelet hyper-responsiveness in dextran sodium sulfate (DSS)-induced murine models. The authors remarked that DSS colitis in wild-type (WT) mouse models resulted in thrombocytosis (with increases in both the mature and immature fractions of platelets) and an increased platelet sensitivity to thrombin, resulting in a prothrombotic phenotype. Notably, this phenotype was absent in IL-6 ^−/−^ mice. To support these experimental findings, the authors demonstrated that exogenous administration of murine IL-6 to WT control and DSS mice promoted and accelerated the formation of light/dye-induced (micro)-thrombi in cremaster arterioles, which the administration of anti-IL-6 antibodies reversed.

### 6.2. Infectious Diseases

Infection with the highly contagious SARS-CoV-2 virus, the causative agent behind the coronavirus 2019 (COVID-19) pandemic, has been associated with an increase in circulating tumour-necrosis factor (TNF) and IL-6, in part contributing to hypercoagulation and platelet dysfunction. Approximately 24% of COVID-19 patients develop systemic thrombocytopenia, with a small percentage developing immune thrombocytopenic purpura (ITP) [195]. Increased serum IL-6 and decreased platelet counts are associated with increased severity and mortality in COVID-19 patients [196]. The disproportionate immune response observed in hospitalised COVID-19 patients is partly due to the development of Cytokine Storm Syndrome (CSS), in which the over-stimulation of alveolar macrophages results in an overwhelming release of inflammatory cytokines, which can ultimately contribute to the development of multi-organ failure (MOF). IL-6 is an essential constituent driving the development of cytokine storm by amplifying this response through CD41/16+ inflammatory macrophage activation [197].

Pre-incubation of COVID-19 patient plasma with whole blood of healthy patients demonstrated platelet hyperactivity and hypercoagulability similar to those observed in vivo. However, when tocilizumab, a humanised anti-human IL-6 receptor antibody, was pre-incubated in the COVID-19 patient’s plasma, this effect was significantly blunted, indicating that IL-6 and IL-6R may contribute to the hypercoagulability observed in clinical settings [198]. Studies have demonstrated that the SARS-CoV-2 spike protein can promote IL-6 trans-signalling through increasing the rate of IL-6R shedding [199]. This increase in sIL-6R is seen in patients across the spectrum, from severely ill to mild cases, and is long-term. However, this seemingly only compromises and overwhelms the IL-6 buffering system in critically ill ICU patients [200].

Curiously, COVID-19 patients’ platelets have also been shown to present with elevated P-selectin with impaired agonist-induced phosphatidylserine exposure and integrin α_IIb_β_3_ activation, suggesting that there are also intrinsic changes that affect platelet responsiveness, likely at the megakaryocyte level [201]. This increase in P-selection may contribute to the formation of platelet–leukocyte aggregates, a significant phenomenon observed in various inflammatory and thrombotic conditions. These aggregates are formed when activated platelets bind to leukocytes, a process often mediated by IL-6, through P-selectin and activated MAC-1 [202]. In COVID-19 patients, the majority of circulating neutrophils appeared to be in the form of platelet–neutrophil aggregates [201]. It is possible that this is in part driven by the increased circulating proinflammatory cytokines that have been shown to be platelet primers, IL-6 being one of said cytokines. It is unknown how much IL-6 individually contributes to these pathologies through platelet priming versus other mechanisms.

Sepsis is defined as a life-threatening organ dysfunction caused by a dysregulated host response to infection. It involves a complex interplay between the immune system and infectious agents, leading to widespread inflammation and potential organ failure [203,204]. The pathophysiology of sepsis includes the release of proinflammatory cytokines, oxygen free radicals, and other mediators that can cause endothelial damage, coagulopathy, and microvascular thrombosis, ultimately resulting in hypoxia and multiple organ dysfunction [205]. IL-6 is a crucial mediator in the inflammatory response during sepsis, produced by various cells, including leukocytes and endothelial cells. Elevated IL-6 levels are associated with the development of acute lung injury (ALI) and multiple organ dysfunction syndrome (MODS), with higher concentrations correlating with increased disease severity as measured by APACHE II (Acute Physiology and Chronic Health Evaluation II) and SOFA (Sequential Organ Failure Assessment) scores, respectively [206]. IL-6 contributes to the dysregulated immune response in sepsis, which can lead to systemic inflammation and organ failure [207]. The concentration of IL-6 has been proposed as a potential diagnostic marker for sepsis and septic shock as well as a viable all-cause mortality predictor, with some case reports documenting an increase of up to 7500 times the normal level [208,209,210]. In sepsis, IL-6 is strongly associated with thrombocytopenia and is a marker of poor prognosis, with complications including dysregulated haemostasis and organ dysfunction [210,211,212]. This is likely due to a decrease in thrombopoiesis combined with increased platelet activation, consumption and increased destruction in the spleen [213].

Disseminated intravascular coagulation (DIC), a rare, complex, and often fatal complication from haematological malignancies or sepsis, is perpetuated by the continual consumption of clotting factors and platelets, typically resulting in widespread thrombosis followed by haemorrhage [214]. Shimizu, et al. [215] noted a substantial increase in IL-6 and TPO, both pleiotropic cytokines capable of driving platelet hyper-responsiveness in sepsis-related DIC patients, and demonstrated that the addition of IL-6 and TPO enhanced the release of soluble CD40L (sCD40L) and platelet-derived microparticles (PDMP), facilitating increased platelet–endothelial and platelet–leukocyte interactions.

## 7. Conclusions

Although platelets lack the membrane-bound IL-6 receptor, they retain the gp130 signalling subunit, which enables IL-6 trans-signalling via the soluble IL-6 receptor. This mechanism permits IL-6 to engage intracellular kinases in platelets, most notably JAK2/STAT3, but also elements of the MAPK and PI3K/Akt pathways. Accumulating evidence indicates that IL-6 triggers a partial, “primed” state rather than outright platelet activation. Previous experimental evidence may have inadvertently obscured the potential of IL-6 to prime platelets due to its ability to drive megakaryocyte maturation, in addition to thrombopoiesis, resulting in increased levels of ‘young’, more active platelets in vivo. The mechanism of IL-6 priming is unique, as it appears to enhance platelet activation in response to collagen/GPVI specifically, but not other agonists such as thrombin. This may be explained by cross-talk at the level of JAK2, the latter of which is involved in collagen, but not thrombin-stimulated platelet function. Clinically, high plasma IL-6 levels correlate with platelet hyperreactivity in diseases marked by chronic inflammation, including COVID-19, autoimmune disorders, and certain malignancies. Experiments using IL-6-blocking or IL-6R-blocking agents (i.e., tocilizumab) suggest that mitigating IL-6 trans-signalling may reduce platelet-driven thrombotic complications without fully compromising normal haemostasis. Nevertheless, further research is needed to clarify whether IL-6 priming consistently manifests as enhanced thrombus formation in vivo and to dissect the relative contributions of JAK2/STAT3 versus other downstream effectors. Current data identify IL-6 as a pivotal, though not sole, link between the inflammatory milieu and platelet-mediated thrombotic events, underscoring its potential as a therapeutic target in hypercoagulable states.

## Figures and Tables

**Table 1:** A non-exhaustive list of platelet primers, associated receptors and downstream signalling pathways.

Compound	Activation Threshold	Receptor(s) Involved	Mechanism	References
Epinephrine	↓	α_2A_	Stimulates Gα_i/z_- and Gα_q_-signalling; activates PI3K/Akt	[50,51]
Gas6	↓	Tyro3, UFO and MerTK	Activates PI3K/Akt	[52,53]
IGF-1	↓	IGF-1R	Activates PI3K/Akt	[54,55]
MMP2	↓	PAR-1 with α_Iib_β_3_ as a cofactor	Cleaves PAR-1 at a non-canonical site, stimulates Gα_q_, Gα_12-13_ and Gα_i/z_ responses; activates PI3K/Akt	[56,57,58]
Ox-LDL	↓	CD36 (via ligation)	Induction of tyrosine phosphorylation and Rho kinase activation	[59,60,61,62,63]
PF4	↓	c-Mpl	Activates JAK2/STAT3 and PI3K/Akt	[64,65,66]
PGE_2_	↑/↓	Primarily EP_3-4_	EP_3_ stimulates Gα_i/z_-signalling (at low PGE_2_ concentrations: priming); EP_4_ stimulates Gα_s_-signalling (at high PGE_2_ concentrations: inhibition)	[67,68,69,70]
sCD40L	↓	CD40	Activates p38 MAPK and NF-κB	[71,72,73]
S1P	↑/↓	S1PR_1,4-5_	S1PR_1_ stimulates Gα_i/z_-signalling (at low S1P concentrations: priming); S1PR_4-5_ stimulates Gα_12-13_-signalling, potentially impairing Gα_q_-signalling (at high concentrations: inhibitory)	[74,75,76]
Succinate	↓	SUCNR1	Stimulates Gα_i/z_- and Gα_q_-signalling	[77,78]
TPO	↓	c-Mpl	Activates JAK2/STAT3, PI3K/Akt and MAPK	[16,79]

↓ indicates the reduction of the activation threshold, ↑ indicates the increase of the activation threshold. cAMP, cyclic adenosine monophosphate; c-Mpl, thrombopoietin receptor; EP_3-4_, prostaglandin EP3-4 receptor; Gas6, growth arrest-specific 6; IGF-1/R, insulin growth-like factor-1/receptor; JAK2, Janus kinase 2; MAPK, mitogen-activated protein kinase; MMP-2, matrix metalloproteinase 2; MerTK, proto-oncogene tyrosine-protein kinase MER; NF-κB, nuclear factor kappa B; Ox-LDL, oxidized low-density lipoprotein; PAR-1, protease-activated receptor-1; PF4, platelet factor 4; PI3K, phosphoinositide 3-kinase; PKC, protein kinase C; PLC, phospholipase C; PGE2, prostaglandin E2; S1P/1, 4-5, sphingosine-1-phosphate/receptor 1, 4-5; sCD40L, soluble CD40 ligand; STAT3, signal transducer and activator of transcription 3; SUCNR1, succinate receptor 1; TP, thromboxane receptor; TPO, thrombopoietin; Tyro3, tyrosine-protein kinase receptor Tyro3; TxA2, thromboxane A2; UFO, tyrosine-protein kinase receptor UFO.

## Data Availability

Not applicable.

## Abbreviations

The following abbreviations are used in this manuscript:

5-HT	5-Hydroxytryptamine (Serotonin)
ADP	Adenosine Diphosphate
Akt	Protein Kinase B
ALI	Acute Lung Injury
APACHE II	Acute Physiology and Chronic Health Evaluation II
APS	Antiphospholipid Syndrome
C-Mpl	Thrombopoietin Receptor
CD40L	CD40 Ligand
CRP	C-Reactive Protein
CSS	Cytokine Storm Syndrome
CXCL	C-X-C Motif Chemokine Ligand
cPLA_2_	Cytosolic Phospholipase A2
DAMPs	Damage-Associated Molecular Patterns
DIC	Disseminated Intravascular Coagulation
EP3-4	Prostaglandin E Receptors 3 and 4
Fcɣ	Fc-Receptor Gamma Chain
Gab1	GRB2-Associated Binding Protein 1
Gas6	Growth Arrest-Specific 6
GPVI	Glycoprotein VI
Grb2	Growth Factor Receptor-Bound Protein 2
GSK-3	Glycogen Synthase Kinase 3
HSC	Hematopoietic Stem Cell
IGF-1	Insulin-Like Growth Factor-1
IL-1β	Interleukin-1 Beta
IL-6	Interleukin-6
IP3	Inositol Triphosphate
ITP	Immune Thrombocytopenia
ITAM	Immunoreceptor Tyrosine-Based Activation Motif
JAK	Janus Kinase
LDBM	Low-Density Bone Marrow
LPS	Lipopolysaccharide
MAPK	Mitogen-Activated Protein Kinase
MEK	Mitogen-Activated Protein Kinase
MODS	Multiple Organ Dysfunction Syndrome
MPO	Myeloperoxidase
MMP2	Matrix Metalloproteinase 2
NF-κB	Nuclear Factor Kappa B
NO	Nitric Oxide
Ox-LDL	Oxidized Low-Density Lipoprotein
PAR	Protease-Activated Receptor
PECAM	Platelet Endothelial Cell Adhesion Molecule
PDK1	3-Phosphoinositide-Dependent Protein Kinase 1
PF4	Platelet Factor 4
PI3K	Phosphoinositide 3-Kinase
PIP2	Phosphatidylinositol 4,5-Bisphosphate
PIP3	Phosphatidylinositol (3,4,5)-Triphosphate
PKC	Protein Kinase C
PLC	Phospholipase C
PLA	Platelet–Leukocyte Aggregate
PGE2	Prostaglandin E2
PRP	Platelet-Rich Plasma
RANTES	Regulated on Activation, Normal T Cell Expressed and Secreted
RAP1b	Ras-Related Protein 1b
RASA3	RAS P21 Protein Activator 3
ROCK	Rho-Associated Protein Kinase
ROS	Reactive Oxygen Species
S1P	Sphingosine-1-Phosphate
S1PR1, 4-5	Sphingosine-1-Phosphate Receptors 1, 4, and 5
SCF	Stem Cell Factor
gp130	Soluble Glycoprotein 130
SHP-2	Src Homology 2 Domain-Containing Phosphatase-2
SLE	Systemic Lupus Erythematosus
SOFA	Sequential Organ Failure Assessment
SOS1	Son of Sevenless Homolog 1
STAT3	Signal Transducer and Activator of Transcription 3
STEMI	ST-Elevation Myocardial Infarction
SUCNR1	Succinate Receptor 1
Syk	Spleen Tyrosine Kinase
TCZ	Tocilizumab
TLR	Toll-Like Receptor
TNF-α	Tumour Necrosis Factor-Alpha
TPO	Thrombopoietin
TP	Thromboxane Receptor
TxA2	Thromboxane A2
TxB2	Thromboxane B2
Tyro3	Tyrosine-Protein Kinase Receptor 3
UFO	Axl Receptor Tyrosine Kinase
VWF	Von Willebrand Factor
